# Combination of coronary CT angiography, FFR_CT_, and risk factors in the prediction of major adverse cardiovascular events in patients suspected CAD

**DOI:** 10.1002/clc.23989

**Published:** 2023-03-01

**Authors:** Shinichi Wada, Yoshitaka Iwanaga, Michikazu Nakai, Yoko M. Nakao, Yoshihiro Miyamoto, Teruo Noguchi, for The NADESICO Study Investigators

**Affiliations:** ^1^ Department of Medical and Health Information Management National Cerebral and Cardiovascular Center Suita Japan; ^2^ Department of Cardiology Sakurabashi Watanabe Hospital Osaka Japan; ^3^ Leeds Institute of Cardiovascular and Metabolic Medicine University of Leeds Leeds UK; ^4^ Department of Cardiology National Cerebral and Cardiovascular Center Suita Japan

**Keywords:** cardiovascular risk factor, computed tomography, diabetes mellitus, fractional flow reserve, major adverse cardiovascular event

## Abstract

**Background:**

To examine the utility of fractional flow reserve by coronary computed tomography (CT) angiography (FFR_CT_) for predicting major adverse cardiovascular events (MACE) in patients with suspected coronary artery disease (CAD).

**Methods:**

This was a nationwide multicenter prospective cohort study including consecutive 1187 patients aged 50−74 years with suspected CAD and had available coronary CT angiography (CCTA). In patients with ≥50% coronary artery stenosis (CAS), FFR_CT_ was further analyzed. The Cox proportional hazards model was used to examine the association of FFR_CT_ and cardiovascular risk factors with incident MACE within 2 years.

**Results:**

Among 933 patients with available information on MACE within 2 years after enrollment, the incidence rate of MACE was higher in 281 patients with CAS than in those without CAS (6.11 vs. 1.16 per 100 patient‐year). In 241 patients with CAS, the Cox proportional hazards analysis showed that FFR_CT_ as well as diabetes mellitus and low high‐density lipoprotein cholesterol level were independently associated with incident MACE. Moreover, the hazard ratio was significantly higher in patients harboring all three factors compared to those harboring 0−2 of the three factors (6.01; 95% confidence interval: 2.77−13.03).

**Conclusions:**

Combinatorial assessment using CCTA for stenosis, FFR_CT_, and risk factors was useful for more accurate prediction of MACE in patients with suspected CAD. Among patients with CAS, those with lower FFR_CT_, diabetes mellitus, and low high‐density lipoprotein cholesterol level were at highest risk for MACE during the 2‐year period following enrollment.

## Graphical abstract

This was a nationwide multicenter prospective cohort study including consecutive 1187 patients aged 50−74 years with suspected coronary artery disease and available coronary computed tomography angiography.

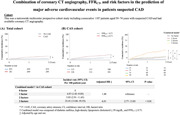

**Abbreviations**
CAC: coronary artery calcification
CAD: coronary artery disease
CAS: coronary artery stenosis
CCTA: coronary computed tomography angiography
CI: confidence interval
FFR_CT_
: fractional flow reserve determined with CCTA
HDL: high‐density lipoprotein
HR: hazard ratio
MACE: major adverse cardiovascular events

## 1 INTRODUCTION

Coronary artery stenosis (CAS) is associated with the future development of cardiovascular events and is therefore important in the evaluation of patients with suspected or known coronary artery disease (CAD).^1^ Coronary computed tomography (CT) angiography (CCTA) is a commonly used high‐utility tool for the noninvasive evaluation of CAS, and CCTA findings inform treatment approaches including percutaneous coronary intervention.^2^ Fractional flow reserve (FFR) determined with CCTA (FFR_CT_), which reflects coronary artery blood flow and reserve capacity, has been recently approved for the clinical evaluation of cardiac ischemia in patients with CAS.^3^, ^4^, ^5^, ^6^ A cutoff FFR_CT_ value of ≤0.8 was demonstrated to indicate reduced capacity of coronary artery flow, which suggested therapeutic intervention and revascularization.^7^ Moreover, a recent meta‐analysis suggested that negative FFR_CT_ was associated with low incidence of adverse events at 12 months compared to positive FFR_CT_ in patients with stable CAD.^8^ However, more accurate and valuable prediction of major adverse cardiovascular events (MACE) using FFR_CT_ is open to consideration. The predictive ability of FFR_CT_ in combination with cardiovascular risk factors for future MACE, which has not yet been elucidated, might be an effective strategy for the management of patients with suspected and known CAD. Therefore, we investigated the utility of a combinatorial approach, including cardiovascular risk factors and CCTA to evaluate stenosis and FFR, in predicting future MACE in patients with suspected CAD. To this end, we used data from the Nationwide Gender‐Specific Atherosclerosis Determinants Estimation and Ischemic Cardiovascular Disease Prospective Cohort (NADESICO) study.^9^

## 2 METHODS

### 2.1 Study design

This study was a subanalysis of the NADESICO study, a prospective, multicenter cohort study that was designed to evaluate sex differences in the association of coronary atherosclerosis including coronary artery calcification (CAC) with MACE. The study protocol was registered with the UMIN Clinical Trials Registry (UMIN‐CTR ID: UMIN000001577) before the release of data to the lead author of the present study. The protocol was approved by the institutional review boards of all participating centers including the National Cerebral and Cardiovascular Center (NCVC) (M20‐029‐9), and written informed consent was obtained from all patients before participation.

### 2.2 Participants

The detailed study protocol was described elsewhere.^9^, ^10^ Briefly, patients included in the present study were consecutively recruited between December 2008 and April 2013 from the cardiology departments of 15 hospitals in Japan participating in the NADESICO study. Follow‐up after enrollment was conducted every year starting from enrollment until March 2020. The inclusion criteria were as follows: age, 50−74 years; suspected CAD in a stable setting; and sufficient indications for plain CT and CCTA. Patients fulfilling the following criteria were excluded: history of myocardial infarction or treatment with percutaneous coronary intervention or coronary artery bypass grafting, history of Kawasaki disease, coronary artery malformation, familial hypercholesterolemia, obviously limited prognosis due to malignant tumors, dialysis, and treatment for serious mental or neurological disorder.

### 2.3 Data collection

Clinical data on diagnostic and therapeutic measures were collected by investigators at each hospital and sent to the NCVC. Hypertension was defined as current use of antihypertensive agents with systolic blood pressure >140 mmHg or diastolic blood pressure >90 mmHg while resting. Diabetes mellitus was defined as self‐reported history of adult‐onset fasting glucose ≥126 mg/dL or use of insulin or oral glucose‐lowering medications. Dyslipidemia was defined as the current use of any lipid‐lowering agents, triglyceride ≥150 mg/dL, low‐density lipoprotein cholesterol ≥140 mg/dL, or high‐density lipoprotein (HDL) cholesterol ≤40 mg/dL in men and ≤50 mg/dL in women. Data on smoking habits and medical history were collected at enrollment by a questionnaire. Laboratory examination included complete blood count, lipid profile, and plasma glucose level.

CT was performed using 64 or more channels with electrocardiography gating according to the Japanese Circulation Society guidelines and institutional protocols. The CT images were digitally transferred to the NCVC and interpreted in a blind fashion by an independent imaging core laboratory using SYNAPSE VINCENT　(FUJIFILM Medical Co., Ltd). A Japan Radiological Society Board‐certified radiologist with extensive experience in coronary CT blinded to all clinical data interpreted the plain CT images using the Agatston CAC scoring method. In CCTA images, all >1.5 mm vessels were assessed for the presence of stenosis and the severity was determined by visual estimation using a percentage of the vessel diameter. The stenosis severity with ≥50% was defined as CAS in the present study. Additionally, FFR_CT_ analysis was performed in patients with CAS. All FFR_CT_ analyses were performed in HeartFlow, Inc. In each patient, the lowest FFR_CT_ value of major coronary arteries, such as right coronary artery, left anterior descending coronary artery, and circumflex branch artery, was defined as the FFR_CT_ value used for all analyses. All FFR_CT_ values lower than 0.50 were defined as an FFR_CT_ value of 0.50.

### 2.4 Follow‐up and outcomes

All patients were evaluated for the presence of MACE. Attending physicians contacted patients who did not visit the hospital more than once a year via telephone or mail. If a patient visited another hospital with CAD, the attending physician inquired about the onset with the hospital. MACE included cardiovascular death, myocardial infarction, late revascularization (>3 months after the indexed CCTA), stroke, hospitalization for unstable angina, heart failure, and aortic disease. Cardiovascular death was defined as death induced by myocardial infarction, heart failure, cardiac arrhythmia, sudden cardiac death, aortic disease, or stroke.

The primary outcome was the ability of FFR_CT_ to predict MACE within 2 years after enrollment based on a previous report.^11^ The secondary outcomes were the ability of CAS alone and in combination with FFR_CT_ and cardiovascular risk factors to predict MACE within 2 years.

### 2.5 Statistical analysis

Continuous data were presented as means ± standard deviation or medians with interquartile ranges, and categorical data were presented as numbers with percentages. Continuous variables between two groups were compared using Student's *t* or the Mann−Whitney *U* test, and categorical variables between two groups were compared using the *χ* ^2^ test. The rate of MACE according to specific FFR_CT_ ranges (≤0.50, 0.51−0.60, 0.61−0.70, 0.71−0.80, and ≥0.81) was examined by the Cochran−Armitage test for trend. The Cox proportional hazards model was used with adjustment for age, sex, and FFR_CT_ value. Logistic regression analysis was used to examine factors associated with low FFR_CT_, wherein the median FFR_CT_ value of the study cohort was used as the cutoff. The cumulative incidence of MACE during the 2‐year period after enrollment for suspected CAD was estimated using the Kaplan−Meier method and compared using the log‐rank test. Statistical significance was defined as a *p* value of less than .05. All statistical analyses were performed using STATA 17 (StataCorp).

## 3 RESULTS

### 3.1 Baseline characteristics of the total cohort

A total of 933 patients (mean age, 66 ± 6 years), including 430 female patients, with suspected CAD who were followed for the occurrence of MACE during the first 2 years were included in the study (Supporting Information: Figure 1). Forty‐seven patients developed MACE for 2 years (Supporting Information: Table 1). Two‐hundred and eighty‐one patients (30.1%) were diagnosed with CAS using CCTA; these patients were older (*p* < .01), were more likely to be male (*p* < .01), and had higher rates of current or past smoking (*p* < .01), hypertension (*p* < .01), and diabetes mellitus history (*p* < .01) compared to patients without CAS. Moreover, the rate of statin use was higher (*p* < .01) and the levels of HDL and total cholesterol were lower in patients diagnosed with CAS using CCTA (*p* < .01) (Supporting Information: Table 2). The Agatston CAC scores were significantly higher in patients with CAS than in those without CAS (*p* < .01).

### 3.2 Baseline characteristics of the CAS cohort

**Figure 1:**
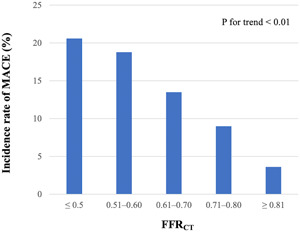
Relationship between FFR_CT_ value and incidence rate of MACE. FFR_CT_, fractional flow reserve derived from coronary computed tomography angiography; MACE, major adverse cardiovascular events.

Data on FFR_CT_ could not be obtained due to technical errors such as motion artifacts in 40 of the 281 patients with CAS (Supporting Information: Figure 1). Of the remaining 241 patients with CAS and available FFR_CT_ data, 28 patients developed MACE within 2 years. The rate of diabetes mellitus was higher in patients with MACE than in those without MACE (*p* < .01). In addition, the glycated hemoglobin (HbA1c) levels were significantly higher and the HDL cholesterol levels were significantly lower in patients with MACE than in those without MACE (*p* < .01) (Table 1). The FFR_CT_ values were significantly lower in patients with MACE than in those without MACE (*p* < .01). Among the cardiovascular risk factors and CCTA‐related values, diabetes mellitus, higher HbA1c, lower HDL cholesterol, higher triglyceride, and lower FFR_CT_ were associated with incident MACE in the Cox proportional hazards model (Table 1). After adjustment for age, sex, and FFR_CT_ value, diabetes mellitus, higher HbA1c, and lower HDL cholesterol were associated with incident MACE (*p* < .01 for all). Additionally, lower FFR_CT_ values were associated with higher MACE incidence within 2 years (*p* for trend, <.01; Figure 1). Moreover, an FFR_CT_ value of ≤0.71 was only associated with male sex and lower HDL cholesterol levels among the cardiovascular risk factors included in the Framingham risk score (Supporting Information: Table 3).

**Table 1:** Univariable and multivariable analyses of patient characteristics associated with MACE in patients with available FFR_CT_ data.

	No MACE (*N* = 213)	MACE (*N* = 28)	*p* Value	Univariable			Multivariable^a^		
				HR	95% CI	*p* Value	HR	95% CI	*p* Value
Age, years (per 1 year)	66 ± 6	67 ± 7	.73	1.01	0.95−1.08	.68			
Female sex	74 (34.7)	6 (21.4)	.16	1.89	0.77−4.65	.17			
Current or past smoker	129 (60.6)	22 (78.6)	.06	2.27	0.92−5.60	.08			
Hypertension	150 (70.4)	22 (78.6)	.37	1.50	0.61−3.71	.38			
Diabetes mellitus	76 (35.7)	21 (75.0)	<.01	4.86	2.07−11.44	<.01	4.52	1.92−10.68	<.01
Dyslipidemia	114 (53.5)	19 (67.9)	.15	1.75	0.79−3.86	.17			
Body mass index ≥25 kg/m^2^	72 (33.8)	10 (35.7)	.84	1.07	0.49−2.31	.87			
Systolic blood pressure, mmHg (per 1 mmHg)	135 ± 17	138 ± 17	.28	1.01	0.99−1.03	.29			
Diastolic blood pressure, mmHg (per 1 mmHg)	77 ± 11	78 ± 11	.58	1.01	0.98−1.05	.58			
HbA1c, % (per 1%)	6.3 ± 1.1	6.9 ± 1.0	<.01	1.36	1.08−1.71	<.01	1.40	1.09−1.80	<.01
Total cholesterol, mg/dL (per 1 mg/dL)	195.6 ± 35.0	187.8 ± 36.2	.27	0.99	0.98−1.00	.27			
HDL cholesterol, mg/dL (per 1 mg/dL)	53.2 ± 13.1	45.6 ± 11.6	<.01	0.95	0.92−0.98	<.01	0.96	0.92−0.99	.01
Triglyceride, mg/dL (per 1 mg/dL)	150.4 ± 87.9	187.9 ± 141.5	.05	1.00	1.00−1.01	.02	1.00	1.00−1.01	.18
LDL cholesterol, mg/dL (per 1 mg/dL)	112.2 ± 30.1	106.1 ± 31.6	.32	0.99	0.98−1.01	.29			
Anti‐coagulant use	10 (4.7)	1 (3.6)	.79	0.77	0.10−5.66	.80			
Statin use	80 (37.6)	9 (32.1)	.58	0.79	0.36−1.74	.56			
CAC Agatston score (per 10)	223.1 [56.6−619.13]	398.68 [115.9−1013.4]	.08	1.00	1.00−1.01	.03	1.00	1.00−1.00	.25
FFR_CT_ (per 0.1)	0.72 [0.60−0.80]	0.60 [0.51−0.71]	<.01	0.61	0.44−0.84	<.01	0.63	0.45‐0.87	<.01

*Note*: Data are presented as means ± standard deviation, numbers (%), or medians [interquartile range].

Abbreviations: CAC, coronary artery calcification; CAS, coronary artery stenosis; CI, confidence interval; FFR_CT_, fractional flow reserve by coronary computed tomography angiography; HbA1c, glycated hemoglobin; HDL, high‐density lipoprotein; HR, hazard ratio; LDL, low‐density lipoprotein; MACE, major adverse cardiovascular events; *N*, number.

^a^ Adjustment was made for age, sex, and FFR_CT_ value.

### 3.3 Risk stratification for MACE in the total and CAS cohorts

**Figure 2:**
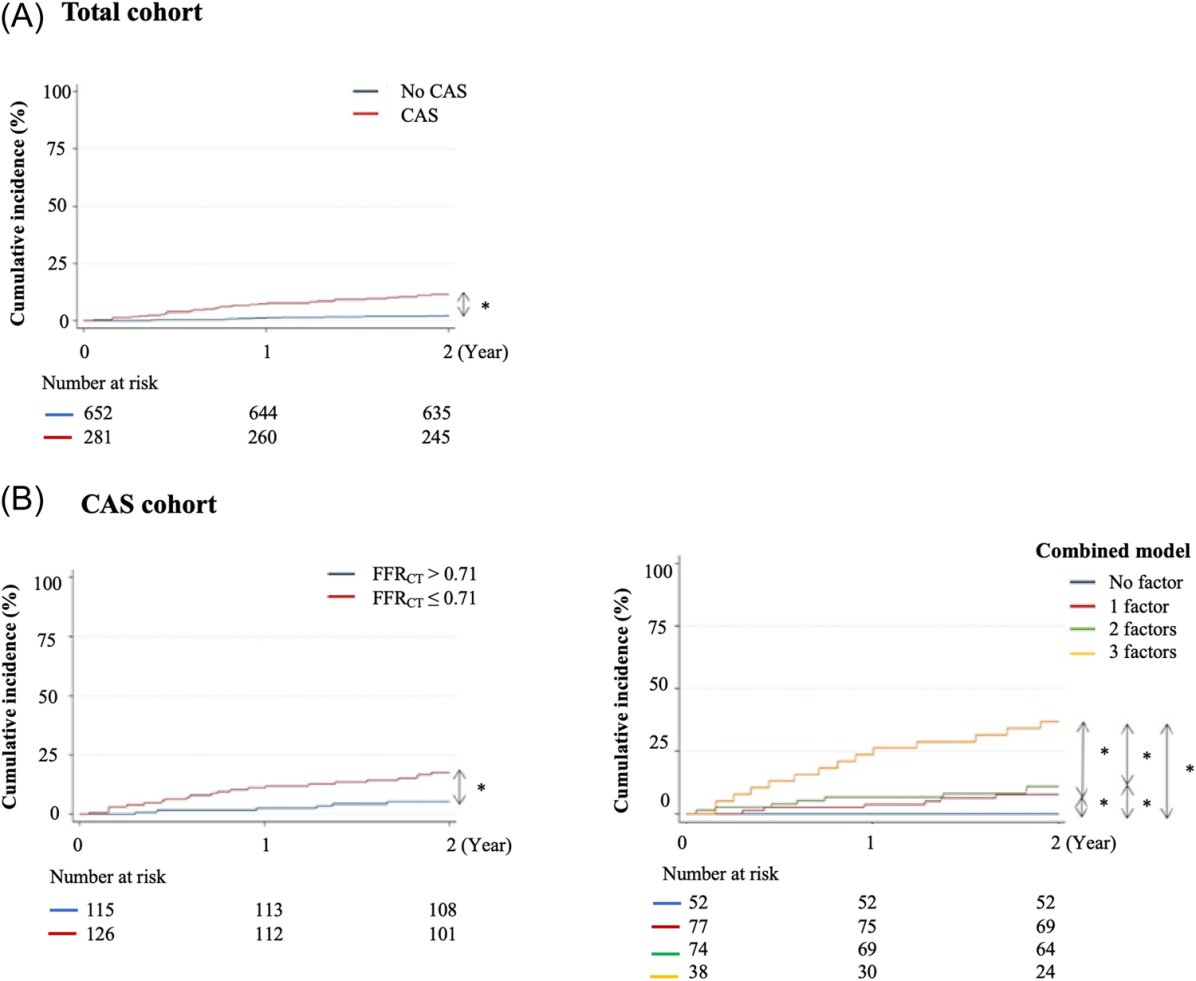
Kaplan−Meier analysis for the incidence of MACE in the (A) total and (B) CAS cohorts. Combined model was composed of diabetes mellitus, high‐density lipoprotein cholesterol ≤50 mg/dL, and FFR_CT_ ≤0.71. *p < .05. CAS, coronary artery stenosis; FFR_CT_, fractional flow reserve derived from coronary computed tomography angiography; MACE, major adverse cardiovascular events.

In the total cohort, the Kaplan−Meier analysis indicated that the incidence of MACE was significantly higher in patients with CAS than in those without CAS (6.11 vs. 1.16 per 100 patient‐year, *p* < .01, log‐rank test; Figure 2A), and the hazard ratio (HR) was 4.65 (95% confidence interval [CI]: 2.49−8.67) after adjustment for age and sex (Table 3).

In the CAS cohort, FFR_CT_, HbA1c, and HDL cholesterol, three continuous variables that were significantly different between the patients with and without MACE (Table 1), were independently associated with the incidence of MACE within 2 years after enrollment (Model 1, Table 2). Moreover, FFR_CT_ ≤0.71, which was based on the median FFR_CT_ value of 0.71 in CAS cohort, history of diabetes mellitus, and low HDL cholesterol (≤50 mg/dL) were independently associated with MACE within 2 years (Model 2, Table 2). In the Kaplan−Meier analysis, the incidence of MACE was significantly higher in patients with FFR_CT_ ≤0.71 than in those with FFR_CT_ >0.71 (Figure 2B). The incidence rate of MACE was higher in patients with FFR_CT_ ≤0.71 than in those with FFR_CT_ >0.71 (9.78 vs. 2.68 per 100 patient‐year), and the HR was 3.31 (95% CI: 1.32−8.32) after adjustment for age and sex (Table 3). In the combined model including the three independent factors in Model 2, patients harboring all three factors had the highest incident rate of MACE (23.41 per 100 patient‐year), and the HR was 6.01 (95% CI: 2.77−13.03) compared with the reference group of patients harboring 0−2 of the three factors, after adjustment for age and sex (Figure 2 and Table 3). In contrast, patients harboring none of the three factors did not develop MACE during the 2 years after enrollment for suspected CAD.

**Table 2:** Multivariable analysis of predictive factors for MACE.

	HR	95% CI	*p* Value
Model 1			
FFR_CT_ (per 0.1)	0.65	0.47−0.90	.01
HbA1c (per 1.0%)	1.34	1.03−1.75	.03
HDL cholesterol (per 1 mg/dL)	0.96	0.93−0.99	.02
Model 2			
FFR_CT_ ≤ 0.71 (median)	2.63	1.05−6.58	.04
Diabetes mellitus	4.05	1.71−9.57	<.01
Low HDL cholesterol (≤50 mg/dL)	2.44	1.02−5.82	.045

Abbreviations: CI, confidence interval; FFRCT, fractional flow reserve by coronary computed tomography angiography; HbA1c, glycated hemoglobin; HDL, high‐density lipoprotein; HR, hazard ratio; MACE, major adverse cardiovascular events.

**Table 3:** Univariable and multivariable analyses of predictive models for MACE.

	Incident rate (95% CI) per 100 patient‐year	Univariable			Multivariable^b^		
		HR	95% CI	*p* Value	HR	95% CI	*p* Value
Total cohort (*N* = 933)							
No CAS	1.16 (0.70−1.93)	1.00	Reference	–	1.00	Reference	–
CAS	6.11 (4.32−8.65)	5.23	2.83−9.66	<.01	4.65	2.49−8.67	<.01
CAS cohort (*N* = 241)							
FFR_CT_ model							
FFR_CT_ > 0.71	2.68 (1.20−5.97)	1.00	Reference	–	1.00	Reference	–
FFR_CT_ ≤ 0.71	9.78 (6.44−14.85)	3.61	1.46−8.91	<.01	3.31	1.32−8.32	.01
Combined model^a^							
0 factor	0	1.00	Reference	–	1.00	Reference	–
1 factor	4.07 (1.83−9.05)						
2 factors	5.83 (2.91−11.65)						
3 factors	23.41 (13.86−39.53)	6.38	3.04−13.39	<.01	6.01	2.77−13.03	<.01

Abbreviations: CAS, coronary artery stenosis; CI, confidence interval; FFRCT, fractional flow reserve by coronary computed tomography angiography; HR, hazard ratio; MACE, major adverse cardiovascular events; *N*, number.

a Combined model included diabetes mellitus, high‐density lipoprotein cholesterol ≤50 mg/dL, and FFR_CT_ ≤ 0.71.

^b^ Adjustment was made for age and sex.

## 4 DISCUSSION

In the present study, the prognostic utility of FFR_CT_ was examined in 933 patients with suspected CAD and CCTA. Our analyses revealed that CAS (stenosis severity with ≥50%) based on CCTA was a predictive factor for the development of MACE, in agreement with previous reports.^12^ We also found that an FFR_CT_ value of ≤0.71 was also a predicting factor for MACE within 2 years in patients with CAS. Furthermore, the impact was enhanced following combination with other risk factors including history of diabetes mellitus and lower HDL cholesterol level.

### 4.1 CCTA in patients suspected CAD

CCTA is recommended as a noninvasive approach for patients with symptomatic chest pain and intermediate CAD risk. Several randomized trials have demonstrated that CCTA has similar or better diagnostic ability and prognostic outcomes compared with standard‐of‐care noninvasive testing.^13^ However, there are limitations in specificity and physician agreement with CCTA in patients with moderate‐severe coronary atherosclerosis with >50% stenosis.^14^ For example, CAC significantly reduces the diagnostic specificity and overall accuracy of CCTA.^15^ Thus, further stratification and refinement in diagnostic and prognostic assessments are necessary, and invasive coronary angiography and invasive FFR are often performed. As a noninvasive test following CCTA, FFR_CT_ assesses functional severity by utilizing computational fluid dynamics to calculate coronary blood flow, and exhibits good correlation with invasive FFR.^16^ The utility of FFR_CT_ has been demonstrated in several studies of patients with suspected CAD and CAS, and FFR_CT_ ≤0.80 was used as a predictive indicator with functional significance.^16^, ^17^, ^18^ Data on clinical outcomes in association with FFR_CT_ are limited compared to the diagnostic data. Therefore, the FFR_CT_ analysis was performed in patients with CAS determined using CCTA among a cohort of patients with suspected CAD in the present study, and the utility of FFR_CT_ in predicting downstream clinical outcomes was examined. Since all patients in the CAS cohort had moderate or severe CAS, the incidence rate of MACE within 2 years after enrollment was higher than that reported in previous studies in which patients with mild or moderate CAS were registered for FFR_CT_.^13^ In addition, the rate of MACE was higher in patients with CAS and an FFR_CT_ value of ≤0.71 than in those with CAS and an FFR_CT_ value of >0.71, supporting the utility of FFR_CT_ in addition to CCTA for the improvement of MACE prediction. The difference in the best cutoff FFR_CT_ value (0.71 vs. 0.80) among the studies may be explained by patient characteristics including CAS severity.

### 4.2 FFR_CT_ as a prognostic factor

Male sex and low HDL cholesterol level were independently associated with an FFR_CT_ value of ≤0.71, consistent with a report by Fairbairn et al. which showed that FFR_CT_ values were higher in women than in men with comparable degree of CAS.^19^ This finding might be explained by the higher likelihood of impaired coronary microvascular reactivity to adenosine in females than in males, resulting in higher FFR.^20^ Sex difference in MACE incidence, which can be associated with clinical outcomes, was not observed among the patients with CAS in the present study.

A recent meta‐analysis suggested that the risk of adverse events during the 12‐month period after diagnosis of stable CAD was lower in patients with negative FFR_CT_ than in those with positive FFR_CT_.^8^ In addition, several trials were conducted to further improve the accuracy of outcome prediction using FFR_CT_. For example, Wang et al. reported using multiple CCTA parameters, including CCTA stenosis ≥50%, low‐attenuation plaque, positive remodeling, napkin‐ring sign, lipid plaque volume proportion, and FFR_CT_ ≤0.83, were independent risk factors for MACE; subsequently, an improved nomogram was created using these independent risk factors.^21^ Examining whether adding cardiovascular risk factors to FFR_CT_ improved the prediction of MACE in patients with CAS was a novel aspect of the present study. The evaluation of patients with CAS using FFR_CT_, with additional assessment for diabetes mellitus and HDL cholesterol levels, was useful in improving the accurate prediction of MACE. Although some reports referred to the possibility that diabetes mellitus might directly interfere with the FFR_CT_ results,^22^ diabetes mellitus, and HbA1c were independently associated with MACE in the Cox proportional hazards model including FFR_CT_ in the present study. Low HDL cholesterol, which was independently associated with positive FFR_CT_, was also associated with MACE, at least in part independently of FFR_CT_. The mechanism is not fully clear, and further investigation for the association is necessary.^23^ Assessment of diabetes mellitus and HDL cholesterol in addition to FFR_CT_ should be considered to predict future clinical outcomes in patients with CAS, and careful follow‐up or invasive interventions may be effective in patients harboring all three risk factors.

### 4.3 Limitation

One of the limitations of the present study is the small number of MACE which might impact the findings, and analysis of larger cohorts are necessary to clarify the utility of combination assessment with FFR_CT_ and risk factors for improved prediction of future MACE. Second, some patients with CAS, in whom FFR_CT_ could not be analyzed because of technical issues including poor image quality, were excluded in the present study. Although there was no obvious difference in patient characteristics between the patients with and without available FFR_CT_ results in the present study (data not shown), how to perform risk stratification in patients without available FFR_CT_ results remains unclear. Finally, FFR_CT_ was not performed in patients without CAS in the present study. The rate of positive FFR_CT_ is low in patients without CAS, and the benefit of FFR_CT_ is considered small in clinical practice from the viewpoint of cost‐effectiveness, which should be verified.

## 5 CONCLUSION

The combination of CCTA for stenosis, FFR_CT_, and risk factors was useful for a more accurate prediction of MACE in patients with suspected CAD. Patients with lower FFR_CT_ value, diabetes mellitus, and low HDL cholesterol levels exhibited the highest risk for 2‐year MACE among patients with CAS.

## AUTHOR CONTRIBUTIONS

*Study concept and design*: Shinichi Wada, Yoshitaka Iwanaga, and Teruo Noguchi. *Data curation*: Shinichi Wada, Yoko M. Nakao, and Michikazu Nakai. *Analysis and interpretation of data*: Shinichi Wada, Michikazu Nakai, and Yoshitaka Iwanaga. *Contribution to the interpretation of the results*: Yoshihiro Miyamoto and Teruo Noguchi.

## CONFLICT OF INTEREST STATEMENT

The authors declare no conflict of interest.

## Supporting information

Supporting information.

## Data Availability

The data sets generated analyzed during the current study are available from the corresponding author on reasonable request.
