# Supplementary material for: Combination of coronary CT angiography, FFRCT, and risk factors in the prediction of major adverse cardiovascular events in patients suspected CAD
Source: Clin Cardiol. 2023 Mar 1;46(5):494–501. doi: 10.1002/clc.23989 (PMC10189082; doi:10.1002/clc.23989)
Supplement: Supplementary file 1 — Supporting information. [file CLC-46-494-s001.pdf]

Supplementary Appendix

List of investigators in the NADESICO study

Masanao Naya (Hokkaido University Hospital, Sapporo, Japan)
Masafumi Watanabe (Yamagata University Hospital, Yamagata, Japan)
Tetsu Watanabe (Yamagata University Hospital, Yamagata, Japan)
Satoshi Yasuda (Tohoku University Hospital, Sendai, Japan)
Michihiro Yoshimura (Jikei University School of Medicine, Tokyo, Japan)
Makoto Kawai (Jikei University School of Medicine, Tokyo, Japan)
Tomonori Okamura (Keio University School of Medicine, Tokyo, Japan)
Hiroaki Naito (Nissei Hospital, Osaka, Japan)
Masahiro Higashi (National Hospital Organization Osaka National Hospital, Osaka, Japan)
Hideaki Morita (Osaka Medical College, Takatsuki, Japan)
Kunihiro Nishimura (National Cerebral and Cardiovascular Center, Suita, Japan)
Yasuhide Asaumi (National Cerebral and Cardiovascular Center, Suita, Japan)
Hiroyuki Miura (National Cerebral and Cardiovascular Center, Suita, Japan)
Yutaka Furukawa (Kobe City Medical Center General Hospital, Kobe, Japan)
Hiroyuki Tsutsui (Kyusyu University Hospital, Fukuoka, Japan)
Tomohiro Kawasaki (Shin Koga Hospital, Kurume, Japan)
Mitsuru Oishi (Kagoshima University Hospital, Kagoshima, Japan)
Masaaki Miyata (Kagoshima University Hospital, Kagoshima, Japan)
Hitonobu Tomoike (NTT Research, Inc., MEI Lab, Tokyo, Japan)
Atsushi Kohno (Kobe University Hspital, Kobe, Japan)
Tetsuya Fukuda (National Cerebral and Cardiovascular Center, Suita, Japan)
Tatsuya Nishii (National Cerebral and Cardiovascular Center, Suita, Japan)
Hisashi Makino (National Cerebral and Cardiovascular Center, Suita, Japan)
Kenji Urano (SoftBank Inc.)
Kanata Ara (SoftBank Inc.)
Yuichi Imai (SoftBank Inc.)
Takayuki Suzuki (SoftBank Inc.)
Junko Masue (National Cerebral and Cardiovascular Center, Suita, Japan)
Yoko Sumita (National Cerebral and Cardiovascular Center, Suita, Japan)
Yusuke Sasahara (National Cerebral and Cardiovascular Center, Suita, Japan)

Supplementary Table 1. Number of major adverse cardiac events

	Total case (N = 933)	Case with CAS (N = 281)	Case with FFR_{CT}≤0.71 (N = 126)	Case with 3 factors (N = 38)
Cardiovascular death	2	1	0	0
Myocadiac infarction	1	0	0	0
Unstable Angina	6	2	2	2
Stroke	3	2	2	2
Heart failure	3	1	0	0
Aortic disease	5	2	1	1
Late revascularization	27	24	17	9
Total MACE	47	32	22	14

CAS, coronary artery stenosis; FFR_{CT}, fractional flow reserve derived from coronary computed tomography angiography; MACE, major adverse cardiac events; N, number.

Three factors included diabetes mellitus, high-density lipoprotein cholesterol ≤ 50 mg/dL, and FFRCT ≤ 0.71.

Supplementary Table 2. Patient characteristics according to CAS

	No CAS (N = 652)	CAS (N = 281)	P value
Age, years	64 ± 7	66 ± 6	<0.01
Female sex	334 (51.2)	96 (34.2)	<0.01
Current or past smoking	305 (46.8)	175 (62.3)	<0.01
Hypertension	358 (54.9)	199 (70.8)	<0.01
Diabetes mellitus	124 (19.0)	118 (42.0)	<0.01
Dyslipidemia	319 (48.9)	155 (55.2)	0.08
Body mass index ≥ 25 kg/m^2	224 (34.4)	98 (34.9)	0.88
Systolic blood pressure, mmHg	134 ±18	136 ± 17	0.29
Diastolic blood pressure, mmHg	79 ± 12	77 ± 11	0.09
HbA1c, %	5.9 ± 0.8	6.4 ± 1.1	< 0.01
Total cholesterol, mg/dL	204.8 ± 33.9	194.6 ± 35.9	< 0.01
HDL cholesterol, mg/dL	58.1 ± 14.4	52.2 ± 13.4	< 0.01
Triglyceride, mg/dL	147.0 ± 86.8	153.5 ± 94.2	0.31
LDL cholesterol, mg/dL	116.7 ± 28.4	111.6 ± 31.3	0.02
Anti-coagulant use	43 (6.6)	12 (4.3)	0.17
Statin use	176 (27.0)	105 (37.4)	< 0.01
CAC Agatston score	4.15 [0–56.95]	236.2 [59.2–693.1]	< 0.01

Data are presented as means ± standard deviation, numbers (%), or medians [interquartile range]. CAC, coronary artery calcification; CAS, coronary artery stenosis; HbA1c, hemoglobin A1c; HDL, high-density lipoprotein; LDL, low-density lipoprotein; MACE, major adverse cardiovascular events; N, number

Supplementary Table 3. Multivariable analysis of predictive factors for $FFR_{CT} \leq 0.71$

	OR	95% CI	P value
Age (/1 year)	1.02	0.97–1.07	0.40
Male sex	2.81	1.52–5.16	< 0.01
Diabetes mellitus	1.60	0.91–2.84	0.11
Current smoking	0.60	0.26–1.37	0.22
BP category			
sBP < 120 mmHg and dBP < 80 mmHg	1.00	Reference	Reference
sBP 120–129 mmHg or dBP 80–84 mmHg	0.56	0.21–1.47	0.24
sBP 130–139 mmHg or dBP 85–89 mmHg	0.76	0.31–1.85	0.54
sBP 140–159 mmHg or dBP 90–99 mmHg	0.95	0.39–2.28	0.91
sBP 160 ≤ mmHg or dBP 100 ≤ mmHg	0.61	0.20–1.89	0.39
Total cholesterol (/ 1mg/dL)	1.00	0.99–1.01	0.45
HDL cholesterol (/ 1mg/dL)	0.96	0.94–0.99	< 0.01

BP, blood pressure; CI, confidence interval; dBP, diastolic blood pressure; FFR_{CT}, fractional flow reserve derived from coronary computed tomography angiography; HDL, high-density lipoprotein; OR, odds ratio; sBP, systolic blood pressure

Predictive factors were selected based on the Framingham risk score.

Supplementary Figure 1. Patient selection in this study

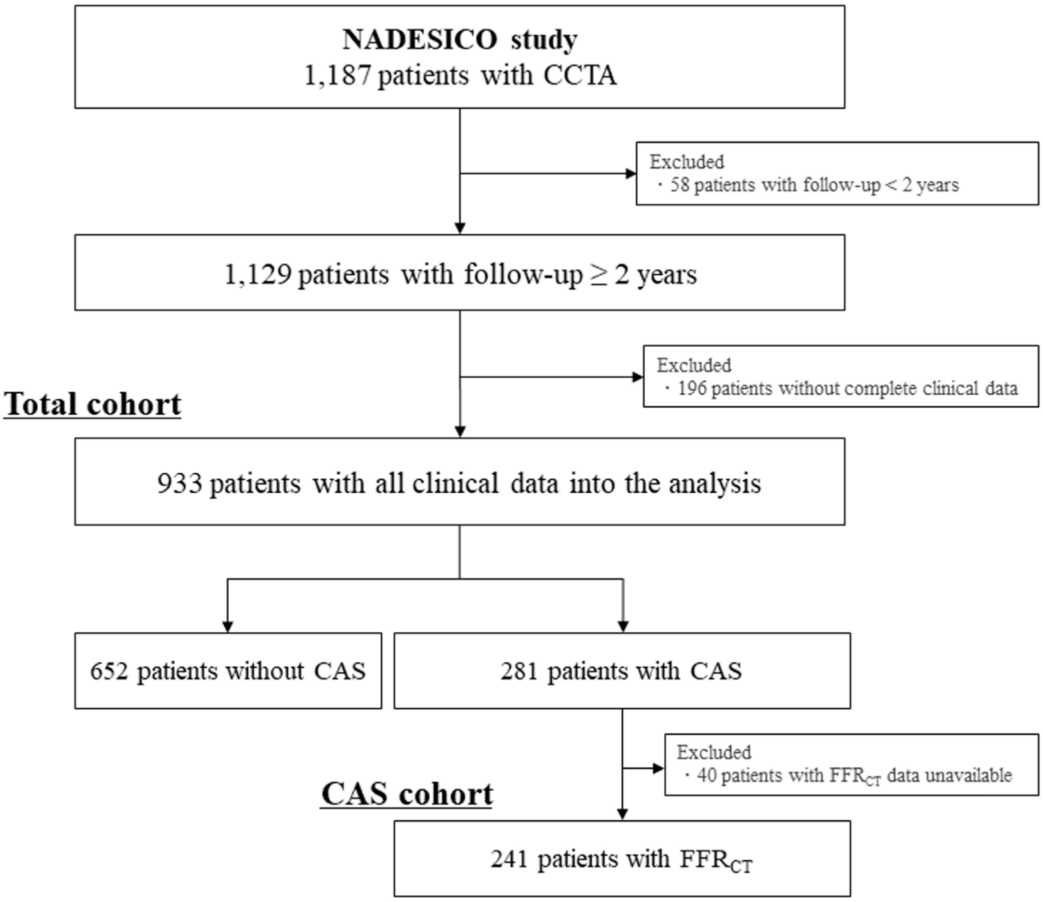

CAS, coronary artery stenosis; CCTA, coronary computed tomography angiography; FFR_{CT}, fractional flow reserve derived from coronary computed tomography angiography; MACE, major adverse cardiovascular events
